# Potential Utility of Circulating MicroRNA-483 as a Biomarker for IGF-II–Associated Non–Islet Cell Tumor Hypoglycemia

**DOI:** 10.1210/clinem/dgae879

**Published:** 2024-12-17

**Authors:** Mikiko Okazaki-Hada, Mototsugu Nagao, Akira Asai, Miki Okada-Iwabu, Naomi Hizuka, Lena Eliasson, Hitoshi Sugihara, Izumi Fukuda, Masato Iwabu

**Affiliations:** Department of Endocrinology, Metabolism and Nephrology, Graduate School of Medicine, Nippon Medical School, Tokyo 113-8603, Japan; Department of Endocrinology, Metabolism and Nephrology, Graduate School of Medicine, Nippon Medical School, Tokyo 113-8603, Japan; Islet Cell Exocytosis, Department of Clinical Sciences Malmö, Lund University, 214 28 Malmö, Sweden; Department of Endocrinology, Metabolism and Nephrology, Graduate School of Medicine, Nippon Medical School, Tokyo 113-8603, Japan; Department of Biochemistry, Kagawa University Faculty of Medicine, Kagawa 761-0793, Japan; Tokyo Women's Medical University, Shinjuku-ku, Tokyo 162-8666, Japan; Islet Cell Exocytosis, Department of Clinical Sciences Malmö, Lund University, 214 28 Malmö, Sweden; Department of Endocrinology, Metabolism and Nephrology, Graduate School of Medicine, Nippon Medical School, Tokyo 113-8603, Japan; Department of Endocrinology, Metabolism and Nephrology, Graduate School of Medicine, Nippon Medical School, Tokyo 113-8603, Japan; Department of Endocrinology, Metabolism and Nephrology, Graduate School of Medicine, Nippon Medical School, Tokyo 113-8603, Japan

**Keywords:** IGF-II, MicroRNA, Non-islet cell tumor hypoglycemia

## Abstract

**Context:**

In most cases of non–islet cell tumor hypoglycemia (NICTH), high molecular weight forms of insulin-like growth factor II, commonly referred to as big IGF-II, cause hypoglycemia. MicroRNA-483 (miR-483), encoded within an intron of *IGF2*, has been suggested to be coexpressed with IGF-II.

**Objective:**

The aim of this study is to demonstrate the utility and reliability of circulating miR-483 as a biomarker for diagnosis and therapeutic outcome of NICTH.

**Methods:**

Sera from 145 cases of suspected NICTH, and postoperative sera from 25 surgical cases of confirmed NICTH were subjected to Western blot analysis and enzyme-linked immunosorbent assay for IGF-II and quantitative polymerase chain reaction analysis for miR-483-5p and -3p. Tissue miR-483 expression levels were compared between resected solitary fibrous tumors (SFTs) and their surrounding margins from 11 surgical cases.

**Results:**

NICTH was confirmed in 100 out of 145 cases based on the detection of big IGF-II in their sera. Receiver operating characteristic curve analysis revealed that serum miR-483-5p had a better diagnostic ability for NICTH than serum IGF-II or the classical diagnostic marker the IGF-II to IGF-I ratio. Notably, serum miR-483-5p levels decreased significantly with the disappearance of big IGF-II after surgical tumor resection. Tissue miRNA-483-5p and -3p expression levels were significantly higher in resected SFT tissues than in their surgical margins.

**Conclusion:**

Circulating miR-483-5p, derived from IGF-II-producing tumors, appears to be a more reliable biomarker for diagnosis and therapeutic outcome of NICTH than IGF-II or the IGF-II to IGF-I ratio. These findings highlight the clinical utility of miR-483-5p in the management of NICTH.

Hypoglycemia caused by extrapancreatic tumors is classified as non–islet cell tumor hypoglycemia (NICTH), which is the second most common cause of spontaneous hypoglycemia after insulinoma (1). In the sera of patients with NICTH, high molecular weight forms of insulin-like growth factor II (IGF-II), referred to as big IGF-II, are frequently detected (2, 3). Production of big IGF-II is due to incomplete enzymatic processing of pro-IGF-II to mature, normal-sized IGF-II in tumors. Big IGF-II has increased bioactivity to induce insulin signaling compared with mature IGF-II, resulting in spontaneous hypoglycemia (3, 4). Despite the clear pathophysiology, the diagnosis of NICTH is a laborious manual process. The most reliable method for diagnosing NICTH is Western blot analysis of sera to demonstrate the presence of big IGF-II. However, Western blotting is not commonly used in general clinical practice. Alternatively, serum IGF-II levels can be measured using an enzyme-linked immunosorbent assay (ELISA) kit, but due to the rarity of the disease, no clinically validated IGF-II assay is available; therefore, the measurement must be performed with an ELISA kit for research use. Moreover, the ELISA cannot distinguish differences in the molecular weight of IGF-II (ie, between big IGF-II and mature, normal-sized IGF-II). Furthermore, in most NICTH cases, serum IGF-II levels remain within the normal range even if measured. Therefore, confirming an increased ratio of the IGF-II to IGF-I (IGF-II/IGF-I) is necessary, as it indicates suppression of IGF-I production due to the increased bioactivity of IGF-II (5). A ratio >10 is considered a realistic diagnostic marker for NICTH, but false positive or negative results can be seen in malnutrition, or sepsis and renal failure (2, 6). Therefore, a simpler and more straightforward diagnostic method for NICTH is highly desirable.

MicroRNAs (miRNAs) are small noncoding RNAs that serve as post-transcriptional gene expression regulators of protein-encoding genes, and more than 2300 different miRNAs exist in human cells (7). Since miRNAs have cell- or tissue-specific expression patterns and are stable in the circulation, blood miRNA profiles have been proposed as potential biomarker for cancer, autoimmune, inflammatory, and metabolic diseases (8). Among them, the miRNA-483 (miR-483) family is encoded in an intron of the *IGF2* gene and thus suggested to be coexpressed with IGF-II (9, 10). We have previously reported elevated serum miR-483 levels in patients with NICTH, suggesting diagnostic value of miR-483 for NICTH (11). In this study, the number of suspected NICTH cases was increased to 145 to validate diagnostic utility. Serum miR-483 levels were also compared before and after the surgical treatment of IGF-II–producing tumors in 25 cases of confirmed NICTH to further demonstrate its potential as a biomarker for the therapeutic outcome of NICTH. Furthermore, miR-483 expression levels in the resected tumor tissues were compared with those in the surrounding margin tissues from 11 surgical cases to ascertain whether IGF-II-producing tumors serve as a primary source of circulating miRNA-483.

## Materials and Methods

### Samples

Patients meeting the screening criteria were defined as a case of suspected NICTH (Fig. S1 (12)). First, hypoglycemia was clinically confirmed, and drug-induced hypoglycemia was excluded. Second, patients were excluded if they had endogenous hyperinsulinemia, including insulinoma or autoimmune hypoglycemia. Third, hormonal deficiencies such as adrenal insufficiency and growth hormone deficiency were excluded. Finally, the remaining patients were considered as cases of suspected NICTH. Physicians treating patients with suspected NICTH collected blood samples from the patients and the sera were frozen at −20 °C. The frozen sera were then sent to Nippon Medical School Hospital (Tokyo, Japan) for Western blot analysis of IGF-II molecular weight to confirm NICTH. From June 2015 to January 2023, we received 145 frozen sera from 110 clinical institutions in Japan. Patient characteristics, data on tumor pathology, and blood chemistry, including plasma glucose, serum insulin, C-peptide, and IGF-I levels, were also provided from the physicians treating the patients. IGF-II levels in the sera of the 145 patients were measured using an ELISA kit for human IGF-II (Alpco, Salem, NH, USA, RRID:AB_2905546). The sera were also used to analyze miR-483-5p and -3p expression levels by quantitative polymerase chain reaction (qPCR) as described below.

For those cases with confirmed NICTH in which surgical removal of the IGF-II–producing tumor was deemed successful due to the remission of hypoglycemia (n = 25), postoperative sera were collected by the treating physicians within 14 days of surgery and sent to Nippon Medical School Hospital for Western blotting, ELISA, and qPCR analyses. In cases of a solitary fibrous tumor (SFT, n = 11), tumor tissues and surrounding surgical margins were kept in RNAlater Stabilization Solution (Thermo Fisher Scientific Inc., Waltham, MA, USA) by surgeons and sent to Nippon Medical School for Western blotting and qPCR analyses.

All the diagnostic and research scheme of the present study was approved by the Ethics Committee of the Nippon Medical School Hospital (no. B-2021-455 and no. B-2023-701) and conducted in accordance with the principles of the Declaration of Helsinki. All patients gave informed consent to provide their clinical information and sera, including storage for future studies at Nippon Medical School. The opportunity to opt out was guaranteed for the patients.

### Serum miRNA Extraction and qPCR

Serum miRNA was extracted using NucleoSpin miRNA Plasma columns (Macherey-Nagel, Düren, Germany). In accordance with a previous report on standardized methods for circulating miRNA profiling (13), an exogenous miRNA (cel-miR-283-3p) was added as a control miRNA. Subsequently, cDNA was prepared using the TaqMan Advanced miRNA cDNA Synthesis Kit (Thermo Fisher Scientific). Then, qPCR analysis was performed using TaqMan Advanced miRNA Assays (Thermo Fisher Scientific) for hsa-miR-483-5p (478432_mir), hsa-miR-483-3p (478122_mir), and cel-miR-283-3p (478292_mir). The serum concentrations of miR-483-5p and -3p were determined by comparing the Ct values with that of cel-miR-283-3p using the 2^−ΔΔCt^ method.

### Western Blotting for Serum IGF-II

The molecular weight of serum IGF-II was analyzed by Western blotting as previously reported (14, 15). In brief, acid–ethanol treated serum was subjected on a 15% sodium dodecyl sulfate-polyacrylamide gel electrophoresis under nonreducing conditions and blotted onto a polyvinylidene fluoride membrane. After blocking with skim milk, the membrane was incubated with anti-IGF-II (clone S1F2; Merck Millipore, Burlington, MA, USA, RRID:AB_309640), followed by a horseradish peroxidase–labeled secondary antibody (#7076; Cell Signaling Technology, RRID:AB_330924). The immunoreactive proteins were detected using ECL Prime Western Blotting Detection Reagent (Cytiva, Tokyo, Japan).

### Tissue RNA and Protein Isolation for Quantitative PCR and Western Blotting

Total RNA and protein were simultaneously extracted from SFT tissues and their surgical margins (preserved in RNAlater Solution) using PARIS Kit (Thermo Fisher Scientific) according to the manufacturer's instructions. Tissue miRNA levels were measured using TaqMan Advanced miRNA cDNA Synthesis Kit and TaqMan Advanced miRNA Assays as in serum samples. The miRNA levels were normalized with hsa-miR-103a-3p (478253_mir) and hsa-miR-191-5p (477952_mir) as endogenous controls based on a previous report on the normalization of miRNA expression levels in human normal and tumor tissues (14). From the total RNA, *IGF2* mRNA levels were also measured using SuperScript VILO cDNA Synthesis Kit (Thermo Fisher Scientific) and TaqMan Gene Expression Assays (Thermo Fisher Scientific) for *IGF2* (Hs04188276_m1). The *IGF2* expression levels were normalized to *GAPDH* (Hs02786624_g1). Tissue protein samples underwent acid–ethanol treatment and analyzed for IGF-II molecular weight by Western blotting as described above for the serum samples.

### Statistical Analysis

Values are presented as median with 25th to 75th percentiles. Categorical values were compared using the χ^2^ test, and other values were analyzed using the Mann–Whitney U test. Paired samples were compared by the Wilcoxon matched pairs signed rank test. We compared the diagnostic potential of serum miR-483 levels with serum IGF-II levels and IGF-II/IGF-I for NICTH using receiver operating characteristic (ROC) curve analysis followed by the DeLong test using EZR (Jichi Medical University Saitama Medical Center, Saitama, Japan), a graphical user interface for R (R Foundation for Statistical Computing, Vienna, Austria). Cases with IGF-II levels below the sensitivity of the assay were excluded from the ROC curve analysis. Other data were analyzed and visualized using GraphPad Prism 10.0 (GraphPad Software Inc., La Jolla, CA, USA). Statistical significance was set at *P* < .05.

## Results

### Patient Characteristics of NICTH

A representative Western blot image of sera from cases of suspected NICTH is shown elsewhere (Fig. S2 (12)). Big IGF-II was detected in 100 of the 145 sera. These 100 cases were definitively diagnosed as NICTH (big IGF-II group). In contrast, sera from 45 cases showed only mature IGF-II of normal molecular weight (designated as normal IGF-II group). Plasma glucose and serum insulin levels were not significantly different between the 2 groups, but serum C-peptide levels were lower in the big IGF-II group (Table 1). The normal IGF-II group had a significantly lower body mass index. All cases in the big IGF-II group had detectable mass lesions on computed tomography scans compared with 61.3% of the cases in the normal IGF-II group. The most common histology of the mass in the big IGF-II group was SFT in 44% of cases, followed by hepatocellular carcinoma in 13% (Table 2).

**Table 1.** Clinical characteristics of suspected NICTH cases

	Normal IGF-II group (n = 45)		Big IGF-II group (n = 100)		*P* value
	Median (25th to 75th percentiles)	n	Median (25th to 75th percentiles)	n	
Age (years)	76.0 (66.5-83.5)	44	72.0 (60.0-81.0)	99	.124
Male/Female	28/17	45	56/44	100	.483
BMI (kg/m^2^)	17.7 (15.4-22.0)	22	21.5 (19.0-24.3)	60	.039
Plasma glucose (mg/dL)	43.0 (28.0-50.0)	35	36.0 (26.3-44.0)	92	.123
Insulin (pmol/L)	3.1 (1.5-6.9)	25	2.4 (1.2-3.5)	60	.080
C-peptide (nmol/L)	0.123 (0.046-0.204)	24	0.033 (0.023-0.066)	67	<.0001
IGF-I (ng/mL)	18.5 (10.8-67.0)	26	27.0 (19.0-41.0)	85	.159
Mass lesion on CT scans (%)	61.3	31	100	100	<.0001

Categorical values were compared using the χ^2^ test, and other values were analyzed using the Mann–Whitney U test.

Abbreviations: BMI, body mass index; CT, computed tomography; IGF-I, insulin-like growth factor I; NICTH, non–islet cell tumor hypoglycemia.

**Table 2.** Tumor pathologies in big IGF-II group

Tumor pathology (n = 100)	n	%
Solitary fibrous tumor	44	44.0
Hepatic cell carcinoma	13	13.0
Gastrointestinal stromal tumor	11	11.0
Gastric cancer	8	8.0
Colon cancer	3	3.0
Hemangiopericytoma	3	3.0
Liposarcoma	3	3.0
Phyllodes tumor	2	2.0
Lung cancer*^a^*	2	2.0
Neuroendocrine tumor	2	2.0
Ovarian carcinosarcoma	1	1.0
Cancer of unknown origin (adenocarcinoma)	1	1.0
Not known	7	7.0

^*a*^One case was diagnosed based on imaging study.

### Serum IGF-II and miR-483 Levels in NICTH

**Figure 1.**
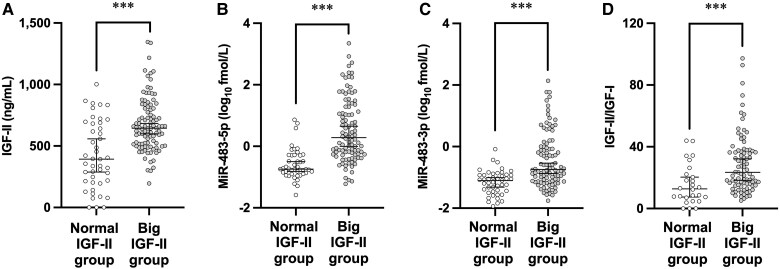
Serum IGF-II, miR-483-5p, miR-483-3p, and IGF-II to IGF-I ratio (IGF-II/IGF-I) in cases of suspected NICTH. Serum IGF-II (A), miR-483-5p (B), and miR-483-3p levels (C) in cases of suspected NICTH (n = 145), and IGF-II/IGF-I (D) calculated in cases where serum IGF-I levels were available (n = 111). These values were compared between the normal and big IGF-II groups. Normal IGF-II group (open circle), n = 45 for IGF-II, miR-483-5p, and miR-483-3p, and n = 26 for IGF-II/IGF-I. Big IGF-II group (gray circle), n = 100 for IGF-II, miR-483-5p, and miR-483-3p, and n = 85 for IGF-II/IGF-I. The central horizontal solid line and the whiskers for each value represent the median and 25th/75th percentiles, respectively. ***P < .001. *P* values were determined using the Mann–Whitney U test. The test was performed using logarithmic values of miR-483-5p and -3p.

**Figure 2.**
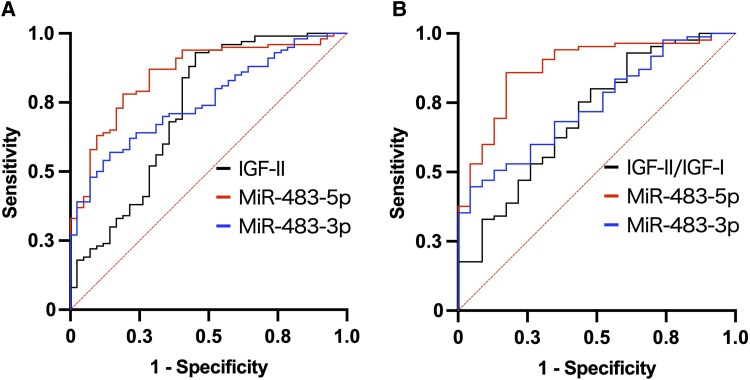
ROC curves of serum IGF-II, miR-483-5p, miR-483-3p, and IGF-II to IGF-I ratio (IGF-II/IGF-I) for the diagnosis of NICTH. (A) ROC curves of serum IGF-II, miR-483-5p, and miR-483-3p for detecting the presence of big IGF-II in cases of suspected NICTH (n = 142). (B) ROC curves of serum IGF-II/IGF-I, miR-483-5p, and miR-483-3p for detecting the presence of big IGF-II in cases of suspected NICTH where serum IGF-I levels were available (n = 108).

Serum IGF-II, miR-483-5p, and miR-483-3p levels were significantly higher in the big IGF-II group than in the normal IGF-II group (Fig. 1A-1C). IGF-II/IGF-I was significantly elevated in the 85 sera of the big IGF-II group compared with the 26 sera of the normal IGF-II group, based on an analysis of 111 cases where IGF-I values were available from medical records. (Fig. 1D). ROC curve analyses demonstrate that the areas under the ROC curves of miR-483-5p and -3p were higher than those of IGF-II or IGF-II/IGF-I (Fig. 2). In particular, miR-483-5p showed significantly higher areas under the ROC curve values than IGF-II or IGF-II/IGF-I (Table 3).

**Table 3.** ROC curve comparison of serum miR-483-5p and miR-483-3p with IGF-II or IGF-II/IGF-I for diagnosing NICTH

	AUC (95% CI)	*P* value
**IGF-II, miR-483-5p, and miR-483-3p (n = 142)**		
IGF-II	0.73 (0.63-0.83)	—
miR-483-5p	0.85 (0.79-0.92)	.045*^a^*
miR-483-3p	0.75 (0.68-0.83)	.668*^a^*
**IGF-II/IGF-I, miR-483-5p, and miR-483-3p (n = 108)**		
IGF-II/IGF-I	0.70 (0.58-0.83)	—
miR-483-5p	0.88 (0.80-0.95)	.0071*^b^*
miR-483-3p	0.74 (0.64-0.84)	.553*^b^*

The diagnosis of NICTH was confirmed by the presence of big IGF-II in the serum. The ROC curves were compared using the DeLong test.

Abbreviations: AUC, area under the curve; IGF-I, insulin-like growth factor I; IGF-II, insulin-like growth factor II; IGF-II/IGF-I, IGF-II to IGF-I ratio; ROC, receiver operating characteristic; NICTH, non–islet cell tumor hypoglycemia.

^*a*^ *P* value vs IGF-II.

^*b*^ *P* value vs IGF-II/IGF-I.

### Surgical Intervention and Serum miR-483-5p Levels

**Figure 3.**
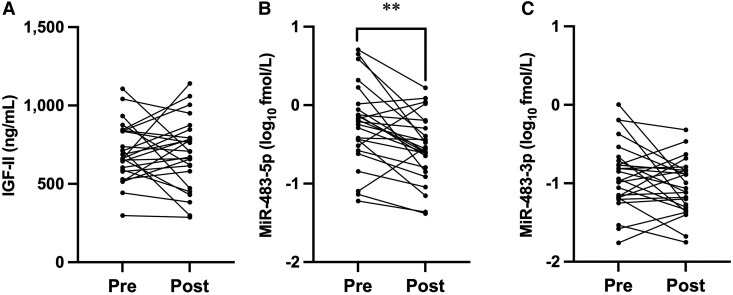
Changes in serum IGF-II, miR-483-5p, and miR-483-3p by surgical intervention. Serum IGF-II (A), miR-483-5p (B), and miR-483-3p levels (C) were compared before and after surgical intervention in 25 cases of confirmed NICTH. Big IGF-II was eliminated by surgical intervention in all 25 cases. **P < .01. *P* values were determined using the Wilcoxon matched pairs signed rank test. The test was performed using logarithmic values of miR-483-5p and -3p.

Spontaneous hypoglycemia disappeared after surgical tumor resection in 25 cases of confirmed NICTH (Table S1 and 2 (12)). In all cases, serum big IGF-II disappeared after the surgical intervention (Fig. S3A (12)), indicating that the resected tumor was the only source of serum big IGF-II. In concordance with the disappearance of big IGF-II, serum miR-483-5p levels decreased significantly after surgery, whereas IGF-II and miR-483-3p levels were not changed (Fig. 3).

### miR-483 in IGF-II–Producing Tumor

**Figure 4.**
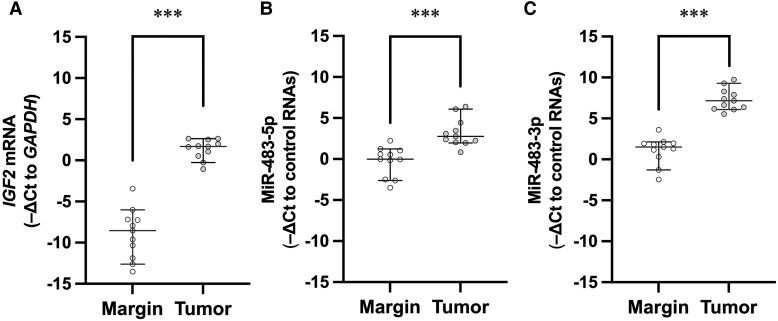
Tissue expression levels of *IGF2* mRNA, miR-483-5p, and miR-483-3p in IGF-II–producing solitary fibrous tumors. Expression levels of *IGF2* mRNA (A), miR-483-5p (B), and miR-483-3p (C) in tumor tissues and the surrounding surgical margins from cases of confirmed NICTH due to solitary fibrous tumors that underwent surgery (n = 11). In these cases, big IGF-II was confirmed by Western blotting in the tumors, but not in the surrounding margins. The central horizontal solid line and the whiskers for each value represent the median and 25th/75th percentiles, respectively. Open circles represent surgical margins (Margin), and gray circles represent tumors (Tumor). ***P < .001. *P* values were determined using the Mann–Whitney U test.

**Figure 5.**
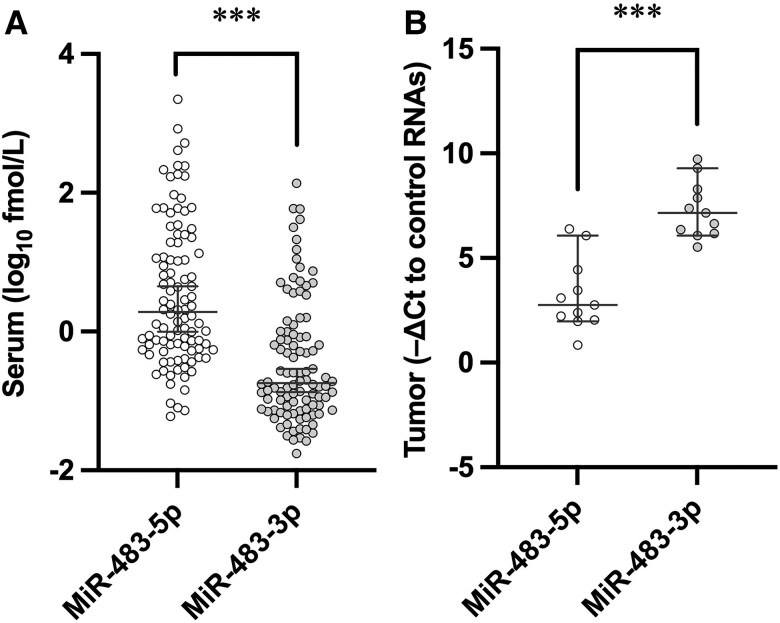
Comparison of miR-483-5p and miR-483-3p expression patterns in serum and tumor tissue of NICTH. (A) Serum miR-483-5p and -3p levels were compared in cases of confirmed NICTH (n = 100). (B) Tissue expression levels of miR-483-5p and -3p were compared in resected solitary fibrous tumors from surgical cases of NICTH (n = 11). The central horizontal solid line and the whiskers for each value represent the median and 25th/75th percentiles, respectively. Open circles represent miR-483-5p, and gray circles represent miR-483-3p. ***P < .001. *P* values were determined using the Mann–Whitney U test. The test was performed using logarithmic values of miR-483-5p and -3p for serum.

Western blot analysis revealed big IGF-II in the resected tumor tissues, but not in the surrounding margins, from the 11 surgical cases of NICTH due to SFT (Fig. S3B (12)). In addition to *IGF2* mRNA, miR-483-5p, and miR-483-3p levels were significantly higher in the resected tumors than in the surrounding margins (Fig. 4). When comparing differential expression of miR-483-5p and -3p in serum and tumor tissue, miR-483-5p was predominant in serum, whereas miR-483-3p was predominant in tumor tissue (Fig. 5).

## Discussion

We have previously proposed that serum miR-483-5p and -3p levels may be potential diagnostic markers for IGF-II–associated NICTH (11). Here, we examined 145 cases of suspected NICTH and confirmed that patients with NICTH show elevated miR-483-5p and -3p levels in serum. In this study, the diagnostic ability of miR-483-5p for NICTH significantly outperformed not only IGF-II but also IGF-II/IGF-I, a classical diagnostic marker for NICTH. In addition, serum miR-483-5p levels were decreased after surgical tumor resection where big IGF-II disappeared, suggesting the potential utility of miR-483-5p as a therapeutic marker also. Furthermore, analysis of resected tumor tissues revealed the overproduction of miR-483 in IGF-II–producing tumors.

Currently, accurate diagnosis of NICTH is based on the molecular weight analysis of serum IGF-II by Western blotting. However, the analytical technique is available only in a limited number of research institutions, which can result in overlooked or delayed diagnoses of NICTH. Serum IGF-II levels are not a reliable diagnostic marker because the levels are within the normal range in most cases of NICTH (5). In this study, only 8 out of 100 (8.0%) cases in the big IGF-II group had serum IGF-II levels above the 95th percentile of healthy adults (>1000 ng/mL). Moreover, serum IGF-I levels are suppressed in NICTH. Therefore, a correction value of IGF-II by IGF-I (ie, IGF-II/IGF-I) >10 is considered a realistic diagnostic marker for NICTH (2, 4, 14, 16). In this study, 78 of 85 (91.8%) cases in the big IGF-II group had IGF-II/IGF-I >10. In contrast, 14 of 23 (60.9%) cases in the normal IGF-II group also had IGF-II/IGF-I >10. These findings suggest that relying solely on IGF-II/IGF-I is not conclusive in the diagnosis of NICTH due to the high false positive rate.

We have proposed the potential utility of serum miR-483-5p and -3p levels as a candidate for another diagnostic marker for NICTH (11). Here, we confirmed elevated miR-483-5p and -3p levels in the patients with NICTH. Prior to this, there had been no better biomarker than IGF-II/IGF-I for the diagnosis of NICTH. Furthermore, miR-483-5p levels decreased in postoperative serum where big IGF-II was eliminated by surgical intervention. These results suggest the clinical use of miR-483-5p or its combination with IGF-II/IGF-I for more efficient diagnosis, therapy, and relapse monitoring in NICTH.

*MiR-483* gene is located within the intron of the *IGF2* gene and encodes 2 mature miRNAs, miR-483-5p and -3p (17). These miRNAs have been shown to be coexpressed with their host gene in hepatocellular carcinoma cells, Wilms tumor, and colorectal cancer tissues (9, 10). Here, in relation to NICTH, we also revealed the simultaneous overexpression of *IGF2* mRNA, miR-483-5p, and miR-483-3p in SFT tissues causing hypoglycemia. Considering the above results that serum miR-483-5p levels were decreased after successful surgical intervention, the IGF-II–producing tumor is most likely a major source of excessive circulating miR-483-5p. In addition, we demonstrated that miR-483-3p was predominant over -5p in tumors, whereas miR-483-5p was predominant in sera. The differential expression patterns of miR-483-5p and -3p between tumor and blood circulation may contribute to the postulated distinctive role of miR-483-5p and -3p in circulation and tissues, respectively. Previous studies reported that miR-483-5p promotes tumor cell migration and invasion (18), whereas miR-483-3p promotes tumor cell proliferation and antiapoptosis (10).

The present study has several limitations. First, miR-483 analysis of 145 cases was performed by using stored sera originally collected for Western blot analysis for the diagnosis of NICTH. Since complete clinical information was not a prerequisite for the Western blot analysis, certain clinical information was missing in some cases. Nevertheless, hypoglycemia was confirmed in all 145 cases and either serum insulin or C-peptide levels were suppressed. Thus, the diagnosis of NICTH would not be invalidated by the absence of other clinical data. In addition, because we are the leading research institution receiving requests for the diagnosis of NICTH nationwide, specimens and patient data collection from more than 100 clinical sites across the country is a major strength of this study. Second, the causes of hypoglycemia in the normal IGF-II group remain elusive. When comparing the clinical characteristics between the normal and big IGF-II groups, the normal IGF-II group had a lower body mass index of less than 18.5 kg/m^2^, which is classified as underweight, suggesting that hypoglycemia may be caused by malnutrition. It is also understandable that IGF-II/IGF-I was increased in some cases in the normal IGF-II group, given that malnutrition lowers IGF-I levels. Furthermore, there are several considerations regarding the clinical application of miR-483 as a biomarker for NICTH. First, establishing specific cutoff values for miR-483 based on tumor type may be necessary for accurate diagnosis. Additionally, although no recurrence of hypoglycemia was observed in this study up to 14 postoperative days, future research should focus on the long-term monitoring of patients to assess the correlation of miR-483 levels with the recurrence or prognosis of NICTH. However, given that recent rapid advances in the field of miRNA research, the measurement of miR-483 will be more available in future clinical settings, increasing its usefulness and validity as a biomarker for NICTH (19, 20).

In conclusion, the present study demonstrated the clinical utility of serum miR-483-5p as a diagnostic and therapeutic outcome marker for IGF-II-associated NICTH. The fact that the diagnostic ability of miR-483-5p exceeds IGF-II/IGF-I, a classical diagnostic marker for NICTH, deserves special attention. Furthermore, analysis of postoperative sera and tumor tissues from patients with NICTH revealed that the excessive circulating miR-483-5p originates from the IGF-II–producing tumors. These results suggest valuable directions for further research, including the accumulation of additional cases and the establishment of a cutoff value for miR-483-5p. Such efforts could enhance the confirmation of diagnosis or recurrence of NICTH and develop more robust diagnostic strategies when combined with other clinical indicators.

## Data Availability

All datasets generated and/or analyzed during the current study are not publicly available but are available from the corresponding author upon reasonable request.

## Abbreviations

ELISA: enzyme-linked immunosorbent assay
IGF: insulin-like growth factor
miR-483: microRNA-483
NICTH: non–islet cell tumor hypoglycemia
qPCR: quantitative polymerase chain reaction
ROC: receiver operating characteristic
SFT: solitary fibrous tumor
